# Effects of weight management by exercise modes on markers of subclinical atherosclerosis and cardiometabolic profile among women with abdominal obesity: a randomized controlled trial

**DOI:** 10.1186/1471-2261-14-82

**Published:** 2014-07-10

**Authors:** Jina Choo, Juneyoung Lee, Jeong-Hyun Cho, Lora E Burke, Akira Sekikawa, Sae Young Jae

**Affiliations:** 1 College of Nursing, Korea University, Seoul, South Korea; 2 Department of Biostatistics, College of Medicine, Korea University, Seoul, South Korea; 3 School of Nursing and Epidemiology and Clinical and Translational Science Institute, University of Pittsburgh, Pennsylvania, USA; 4 Department of Epidemiology, Graduate School of Public Health, University of Pittsburgh, Pennsylvania, USA; 5 College of Arts and Physical Education, University of Seoul, Seoul, South Korea

## Abstract

**Background:**

Few studies have examined the differential effects of weight management by exercise mode on subclinical atherosclerosis. We hypothesized that 3 modes of aerobic, resistance, and combination exercises have differential effects on the flow-mediated dilation (FMD), carotid-femoral pulse wave velocity (PWV), and carotid intima-media thickness (IMT) as well as cardiometabolic profile in weight management.

**Methods:**

A randomized, single-blind trial (ISRCTN46069848) was conducted in Seoul, South Korea between November 2011 and December 2012. Randomized participants were 110 women with abdominal obesity (aerobic group n = 50; resistance group n = 30; combination exercise group n = 30). The treatment period was 12 months with 3-month follow up: A diet-alone intervention for the first 3 months and a diet-plus-exercise intervention for the next 9 months according to exercise modes. The exercise training was designed with an intensity of 50-70% heart rate reserve for 3 days a week in 60-minute-long sessions for 9 months, consisting of 30-minute treadmill and 30-minute bike exercises for aerobic group; upper and lower body exercises with an intensity target of 2 sets and 8–12 repetitions for resistance group; 30-minute resistance and consecutive 30-minute aerobic exercises for combination group.

**Results:**

Ninety-two and 49 participants were analyzed for modified intention-to-treat analysis and per-protocol (PP) analysis, respectively. The 3 exercise modes had no significant differential effects on FMD, PWV, and IMT over time; however, the combination group was found to have significantly lower levels of fasting glucose than the aerobic group (*p* = .034) in the PP analysis. Nevertheless, we observed significant time effects such as reductions in PWV (*p* = .048) and IMT (*p* = .018) in cubic and quadratic trends, respectively, and improvements in body weight, waist circumference, low-density and high-density lipoprotein cholesterol levels, fasting glucose levels, and cardiorespiratory fitness in linear, quadratic, or cubic trends.

**Conclusions:**

For women with abdominal obesity, a combination of aerobic and resistance exercises may be preferable to a single exercise mode for effective glucose control. Regardless of exercise mode, exercise interventions combined with dietary interventions in weight management may be beneficial in reducing the risk of subclinical atherosclerosis and cardiometabolic risk.

## Background

Abdominal obesity is a risk factor for coronary heart disease (CHD). Prospective cohort studies have reported that an increase in waist circumference is significantly associated with CHD incidence and mortality [1]. In addition, abdominal obesity was found to be associated with subclinical atherosclerotic risk, as assessed by endothelial dysfunction [2], aortic stiffness [3], and carotid atherosclerosis [3,4]. Particularly, the risk for CHD associated with increased waist circumference may be prominent in women [1,5-7], and, in this context, the potential for reducing CHD risk may also be significant in women.

Diet-plus-exercise interventions are commonly recommended for enhancing long-term weight management and reducing CHD risk factors in overweight and obese individuals [8,9]. Empirically, exercise-alone interventions have been found to have weaker effects than diet-plus-exercise interventions on weight loss [10,11]. However, exercise training may have beneficial effects on the regression of subclinical atherosclerosis [12]. A few studies have reported that either aerobic or resistance exercise training improved endothelial dysfunction [13,14], aortic stiffness [15,16], and carotid intima-media thickness (IMT) [17]. However, their effects may differ by exercise modes, i.e., aerobic, resistance, or combination exercise, because each mode leads to different patterns of blood flow and levels of pressure on the endothelium and arterial wall [18,19]. Meanwhile, recent studies have reported that a combination of aerobic and resistance exercises may be more effective on improving anthropometric and cardiometabolic profiles than aerobic or resistance exercise alone [20,21]. In particular, combination exercise was reported to be the most efficacious means of decreasing body weight and waist circumference among overweight and obese adults [20,22] and to have an additional beneficial effect on glucose control compared with either aerobic or resistance exercise for those with type 2 diabetes mellitus [21]. However, there is no information regarding whether the 3 exercise modes, i.e., aerobic, resistance, and combination exercises, have differential effects on markers of subclinical atherosclerosis such as endothelial function, aortic stiffness, or carotid IMT as well as cardiometabolic profile among overweight and obese individuals.

The purpose of the study was to test the hypothesis that aerobic, resistance, and combination exercises in a weight management intervention would have significant differential effects on markers of subclinical atherosclerosis, as measured by brachial flow-mediated dilation (FMD), carotid-femoral pulse wave velocity (PWV) and mean IMT levels at the common carotid artery, and cardiometabolic profile among women with abdominal obesity in the Community-based Heart and Weight Management Trial.

## Methods

### Study participants and enrollment procedure

We recruited the study subjects from a community (i.e., Seongbuk-Gu) in Seoul, South Korea. The community was characterized as an urban county with a population size of approximately 490,000 residents. Between November 2010 and November 2011, study participants were recruited via poster, leaflet, telephone, and mass mailing advertisements at the municipal health center, churches, universities, and online communities. Eligible participants were invited to an orientation where they were provided a detailed study overview, asked for consent, and screened for additional inclusion criteria. The inclusion criteria were as follows: healthy women aged between 18 and 65 years, elevated waist circumference (≥85 cm) according to the criteria for abdominal obesity as defined by the Korean Society for the Study of Obesity [23,24], and willingness to be randomly assigned to one of the 3 different exercise modes (i.e., aerobic, resistance, and combination exercise training). The exclusion criteria included current medical conditions such as cardiovascular diseases, diabetes, or cancers; physical limitations restricting exercise ability; current use of hormone therapy; history of participation in a weight loss intervention in the last 1 year; and a weight change in the last 4 weeks prior to participation in our study. Before commencing with the randomization of participants, baseline measurements of body composition (body mass index [BMI] and waist circumference), blood lipids, and fasting glucose were obtained; information regarding sociodemographic and psychosocial variables were obtained through questionnaires. The study was approved by the Institutional Review Board at Korea University (KU-IRB-11-10-A-2). All participants provided written informed consent.

### Study design

**Figure 1:**
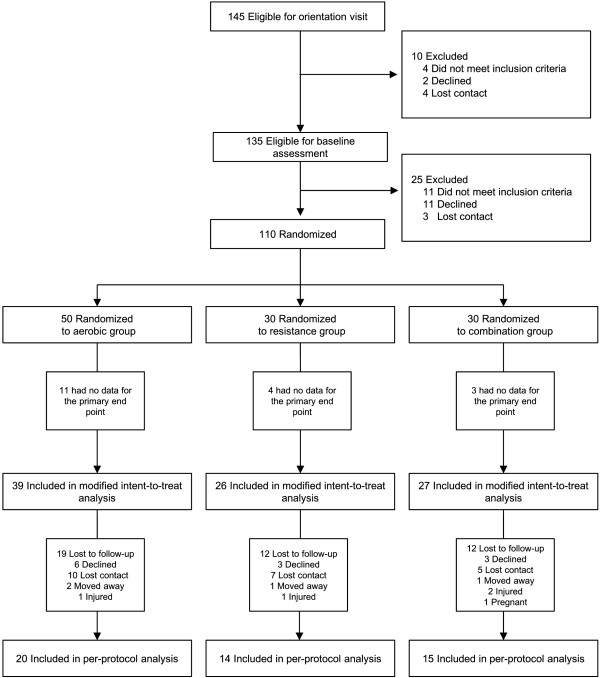
Participant flow chart.

The Community-based Heart and Weight Management Trial (trial registration no. ISRCTN46069848) includes a randomized 12-month intervention with 3 different exercise groups: aerobic training only, resistance training only, and a combination of aerobic and resistance training. The intervention assignment was carried out using a random allocation computer program (n = 50 for aerobic exercise, n = 30 for resistance exercise, and n = 30 for combination exercise) (Figure 1). Initially, the aerobic exercise group (n = 50) was further divided into 2 groups: one group with (n = 30) and the other group without behavioral therapy (n = 20). However, because there were no significant differences in the outcome variables between both the groups, we pooled the participants of those 2 groups into 1 aerobic exercise group (n = 50) for the present study. This study was a single-blinded trial; assessors of all the outcomes were blinded to participant group assignment, and all outcome data were blinded until the completion of final data entry for the 12-month assessment.

### Intervention

**Figure 2:**
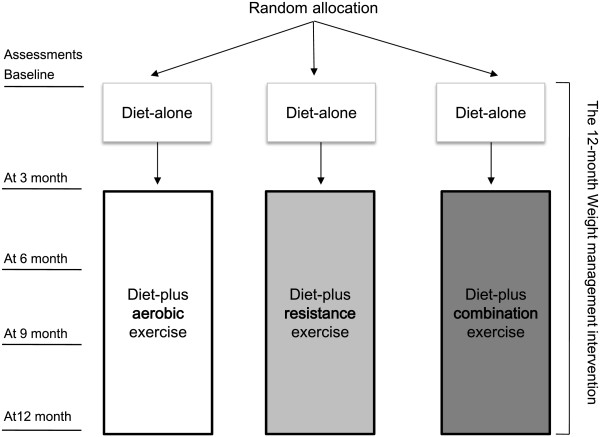
**The 12-month weight management intervention in the present study.** Following random allocation, a diet-alone intervention for the first 3 months and a diet-plus-exercise intervention according to the exercise modes for the next 9 months were implemented over 12 months. The assessments were conducted at baseline, 3, 6, 9, and 12 months.

We conducted 2 consecutive types of interventions: diet-alone vs. diet-plus-exercise interventions in a sequence over a 12-month weight management program (Figure 2), i.e., a diet-alone intervention for the first 3 months and a diet-plus-exercise intervention for the next 9 months according to exercise modes; for the diet-alone intervention, the 3 groups were asked not to attend any exercise training sessions for the first 3 months.

The exercise intervention over the following 9 months was added to the diet-alone intervention: 60-minute exercise sessions were conducted 3 times a week in a public community fitness center under group teaching and supervision of exercise trainers. The aerobic session consisted of 30-minute treadmill exercise and 30-minute bike exercise, with an intensity target of 50–70% of heart rate reserve. The resistance session consisted of both upper and lower body exercises: chest press, lat pull-down, abdominal crunches, back extensions, leg curl, leg extensions, leg press, and calf raise. The load was initially set at 40% and 50% (or 60%) of maximum strength for the upper extremities and lower extremities, respectively. Two sets of 8–12 repetitions were performed during each session. The rest period between each set was less than 90 seconds. The load was progressively increased by 5% every 3 weeks or when subjects could easily perform 15 repetitions. Combination training consisted of 30 minutes of resistance exercise and a consecutive 30-minute session of aerobic exercise (15 minutes on a treadmill and 15 minutes on a bicycle) with 1 set of 8–12 repetitions for resistance exercise and 50–70% of the heart rate reserve for aerobic exercise.

The diet-alone intervention was administered to all participants, with individualized daily calorie intake (1,200 kcal if the weight was <90.5 kg; 1,500 kcal if the weight was ≥90.5 kg) and fat intake goals (≤25% of total calories), taking into account their baseline body weight measurements. Additionally, all the groups except the aerobic group without behavioral therapy (n = 20) received behavioral counseling (12 sessions of group classes over 6 months) and were also taught the use of established behavior change strategies including goal setting and self-monitoring [25].

### Measurements

The primary outcome of the trial was FMD. The secondary outcomes included improvements in other markers of subclinical atherosclerosis (i.e., PWV and carotid IMT), anthropometric profile (i.e., body weight and lean body mass), cardiometabolic profile (i.e., waist circumference, systolic blood pressure, total cholesterol, low-density lipoprotein [LDL] cholesterol, high-density lipoprotein [HDL] cholesterol, triglycerides, and fasting glucose), and cardiorespiratory fitness (i.e., maximal oxygen consumption [VO_2max_]).

All measures were assessed at baseline, 3, 6, and 12 months. Weight was measured after an overnight fast, using the Tanita bioelectrical impedance scale (Tanita Corporation of America, Inc., IL, USA); prior to being weighed, the participants were asked to wear lightweight clothing and take off their shoes. Height was measured using a wall-mounted stadiometer. The BMI was computed as weight (kg)/height (m)^2^. Waist circumference (cm) was measured twice using a Gullick II measuring tape at the midpoint between the lowest rib and the iliac crest; the average of 2 measurements was used.

Blood samples were drawn from the antecubital vein in the morning after a 10-hour overnight fast without taking any current medications, including anti-hypertensive or lipid-lowering medications, when participants visited the municipal health center. Participants who took anti-hypertensive (n = 11) or lipid-lowering medications (n = 7) were instructed to take them during the study period. Samples were analyzed using a biochemical auto-analyzer (Hitachi cobas c501; Hitachi, Japan) at the Department of Clinical Laboratory of the municipal health center, in Seoul, South Korea. Total cholesterol and HDL cholesterol levels were measured by a homogeneous enzymatic colorimetric test. The levels of triglycerides and glucose were measured using the GPO-PAP method and the hexokinase method, respectively. LDL cholesterol level was estimated according to the Friedewald equation [26].

For non-invasive assessment of the FMD, brachial artery dilatation was measured by ultrasonography (ACUSON X 300; Siemens, Mountain View, CA). The brachial artery was imaged in longitudinal sections 5–10 cm proximal to the placement of a blood pressure cuff, just below the antecubital fossa, using a high frequency linear array probe (11.4 MHz). The FMD was measured for 1 minute at baseline and again for 2 minutes after an ischemic stimulus (inflation of a blood pressure cuff around the forearm to 200 mmHg for 5 minutes). The FMD was analyzed using a semi-automated edge detection software system (Brachial Analyzer 5; Medical Imaging Applications, Coralville, IA) to calculate FMD (%) according to the following equation: [(maximum diameter – baseline diameter)/baseline diameter] × 100. A reproducibility (i.e., intraclass correlation coefficient) of 0.95 was achieved within technicians.

Carotid to femoral PWV value was derived to measure central arterial stiffness, using a SphygmoCor®system (AtCor Medical, Sydney, Australia). The distance from the carotid artery sample site to the femoral artery and that from the carotid artery to the suprasternal notch were measured as straight lines with a tape measure. Pulse waves were obtained by applanation tonometry. According to the “foot-to-foot” method, PWV was determined as D/(Δt) (m/s), where Δt is the time difference between carotid-femoral pressure waves, and D is the distance between the 2 arteries. All measurements were taken in duplicate, and the mean values were used for subsequent analysis. Arterial stiffness was measured in accordance with the guidelines of the Clinical Application of Arterial Stiffness, Task Force III. A reproducibility (i.e., intraclass correlation coefficient) of 0.98 was achieved within technicians.

Carotid artery ultrasound imaging was performed using a high-resolution B-mode ultrasound system (ACUSON X 300; Siemens, Mountain View, CA). The IMT was defined as the distance from the leading edge of the lumen-intima interface to the leading edge of the media-adventitia interface of the far wall of the carotid artery. All measurements were made at the end diastole. The carotid IMT of the common carotid artery was determined from a semi-automated measurement obtained 2 cm proximal to the carotid bifurcation. The value for carotid IMT was defined as the mean of the IMT. A reproducibility (i.e., intraclass correlation coefficient) of 0.99 was achieved within technicians.

Cardiorespiratory fitness levels were reported as VO_2max_ values estimated using the Rockport Fitness Walking Test. Participants were administered the 1-mile walk test on an outdoor track in accordance with the protocol of Kline et al. [27].

### Statistical analysis

Sample size calculation was based on Olson et al.’s study [13]. The study showed a decreased mean FMD change of 1.4% (standard deviation [SD], 2.32%) at the 12-month follow-up in the control group and an increased mean FMD change of 2.6% (SD, 3.49%) in the resistance group. We considered two-thirds of mean change difference between groups as an effect size for our study because all of our groups received a diet program. To detect a mean difference in FMD of 2.67% by assuming the SD from Olson et al., between any pairs of the 3 groups in our study, at least 21 subjects per group were needed to ensure an 80% power with the Student’s *t*-test and 2-tailed test at the 5% level of significance. Considering the 30% follow-up loss rate during the study period, we initially planned to randomize a total of 90 subjects to 3 study groups (30 participants per group). However, as mentioned in the Study Design section, the actually allocated sample sizes were 50 participants to aerobic, 30 participants to resistance, and 30 participants to the combination exercise group.

To test the homogeneity of exercise groups in terms of baseline measures for sociodemographic, health-related, subclinical atherosclerosis, anthropometric, cardiometabolic, and cardiorespiratory fitness variables, one-way ANOVA, X^2^ test, and Fisher's exact test were performed. The modified intention-to-treat (ITT) population, defined as randomized subjects with least 1 FMD measure reported across 4 assessment periods, was used to analyze the primary outcome variable. This population consisted of 92 (84%) out of 110 randomized participants (Figure 1). The main effects of exercise group and time as well as the interaction effects between exercise group and time were examined using a generalized estimating equation (GEE) model for repeated measures over time. Covariates adjusted for in the GEE model included baseline values and marital status. Per-protocol (PP) analyses were also conducted; the participants involved in this analysis were defined as a subgroup of participants who met the criteria of 100% follow-up by the fifth assessment over 12 months.

All analyses were performed using STATA 10.0 (StataCorp LP., USA). A 2-tailed p-value of less than 0.05 was considered statistically significant.

## Results

The participants had a mean age of 43.1 years, mean BMI of 28.5 kg/m^2^, and mean waist circumference of 94.8 cm (Table 1). Of all participants, 24.5% had an obesity-related condition such as hypertension or dyslipidemia and 29.1% were post-menopausal. Some of the baseline cardiometabolic profile (i.e., waist circumference and total and HDL cholesterol levels) were outside the reference ranges according to the criteria for abdominal obesity in women as defined by the Korean Society for the Study of Obesity [23], criteria of the National Cholesterol Education Program (NCEP) Adult Treatment Panel III (ATP III) [28], and diagnostic criteria for metabolic syndrome according to the NCEP-ATP III [29]. The mean fitness level, as measured by VO_2max_, was 31.1 mL · kg^−1^ · min^−1^. The participants in the resistance group were highly likely to be married compared to those in the aerobic or combination exercise groups (F = 6.57, p = .037).

**Table 1:** Participants’ characteristics (N = 110)

	**n (%) or mean (SD)**				**F/χ**^**2**^**/Fisher's**	P^*^
	**Total (n = 110)**	**Aerobic (n = 50)**	**Resistance (n = 30)**	**Combination (n = 30)**		
**Sociodemographic characteristics**						
Age, years	43.1 (9.0)	42.2 (9.5)	46.0 (10.0)	41.8 (6.6)	2.16	.120
Married, yes	90 (81.8)	37 (74.0)	29 (97.6)	24 (80.0)	6.57	.037
Education					0.78	.678
Some college or more	66 (60.0)	31 (62.0)	16 (53.3)	19 (63.3)		
High school degree or less	44 (40.0)	19 (38.0)	14 (46.7)	11 (36.7)		
Monthly household income ($)					1.37	.503
<=3,500	61 (55.5)	30 (60.0)	14 (46.7)	17 (56.7)		
> 3,500	49 (44.5)	20 (40.0)	16 (53.3)	13 (43.3)		
Employed, yes	43 (39.1)	19 (38.0)	11 (36.7)	13 (43.3)	0.33	.850
**Health-related characteristics**						
Obesity-related conditions, yes	27 (24.5)	15 (30.0)	8 (26.7)	4 (13.3)	2.91	.233
Post-menopause, yes	32 (29.1)	15 (30.0)	12 (40.0)	5 (16.7)	4.00	.136
Current smoking, yes	6 (5.5)	2 (4.0)	1 (3.3)	3 (10.0)	1.57	.556
Alcohol drinking (twice or more/week), yes	9 (8.2)	4 (8.0)	2 (6.7)	3 (10.0)	0.36	1.000
Anti-hypertensive medication, yes	11 (10.0)	7 (14.0)	4 (13.3)	0 (0.0)	5.22	.101
Lipid-lowering medication, yes	7 (6.4)	0 (0.0)	5 (16.7)	2 (6.7)	8.74	.007
**Markers of subclinical atherosclerosis**						
FMD, %	10.76 (3.457)	11.10 (3.263)	9.94 (3.477)	11.08 (3.723)	1.18	.310
PWV, m/sec	7.86 (1.356)	7.77 (1.615)	7.89 (1.210)	7.99 (1.005)	0.24	.784
IMT, mm	0.67 (0.128)	0.68 (0.118)	0.68 (0.131)	0.66 (0.142)	0.32	.724
**Anthropometric profile**						
Body weight, kg	72.4 (10.55)	72.3 (9.94)	70.8 (10.25)	74.2 (11.86)	0.77	.467
Body mass index, kg/m^2^	28.5 (0.36)	28.5 (0.54)	27.9 (0.58)	29.1 (0.79)	0.79	.456
Lean body mass, kg	46.0 (4.76)	46.0 (4.69)	45.5 (5.11)	46.4 (4.63)	0.25	.776
**Cardiometabolic profile**						
Waist circumference, cm	94.8 (7.80)	94.4 (7.00)	93.8 (7.25)	96.4 (9.46)	0.93	.397
Systolic BP, mmHg	116.5 (13.08)	116.6 (12.97)	117.4 (10.38)	115.6 (15.78)	0.15	.859
Total cholesterol, mg/dL	212.1 (39.74)	214.8 (37.51)	204.5 (45.99)	215.2 (36.81)	0.75	.476
LDL-cholesterol, mg/dL	129.1 (36.39)	130.3 (32.46)	120.2 (42.98)	135.9 (34.75)	1.47	.235
HDL-cholesterol, mg/dL	53.1 (12.61)	53.0 (12.59)	51.6 (15.14)	54.6 (9.78)	0.44	.642
Triglycerides, mg/dL	131.2 (98.40, 73.50)	147.1 (97.10, 205.65)	136.5 (105.05, 177.15)	122.3 (96.13, 146.93)	2.16	.120
Fasting glucose, mg/dL	89.6 (14.45)	90.1 (16.72)	93.6 (14.50)	84.9 (8.05)	2.89	.060
**Dietary intake**^**a**^						
Total energy (kcal/day)	1538.2 (446.01)	1498.9 (441.99)	1552.3 (360.01)	1585.8 (525.19)	0.33	.717
Carbohydrates (g/day)	218.5 (64.91)	217.5 (74.77)	216.8 (40.41)	221.6 (68.26)	0.05	.956
Protein (g/day)	65.4 (25.62)	61.9 (18.10)	66.6 (23.40)	69.6 (35.68)	0.82	.445
Lipid (g/day)	45.6 (18.23)	43.3 (17.53)	46.4 (15.78)	48.4 (21.27)	0.68	.508
**Cardiorespiratory fitness**						
VO_2max_, mL/kg/min	31.1 (6.24)	31.3 (5.80)	30.6 (6.16)	31.3 (7.15)	0.16	.849

BP = blood pressure; FMD = flow mediated dilation; HDL = high-density lipoprotein; LDL = low-density lipoprotein; PWV = pulse wave velocity; IMT = intima media thickness; SD = standard deviation; VO_2max_ = maximal oxygen consumption; Obesity-related conditions include hypertension and dyslipidemia.

^*^P-values from one-way ANOVA, chi-square or Fisher’s exact test as appropriate ^a^Analyzed with the sample size (N = 96).

Table 2 summarizes the results for differential effects of exercise mode on outcome variables over time in weight management, i.e., the interaction effects between exercise groups and time on markers of subclinical atherosclerosis and anthropometric and cardiometabolic profiles on the basis of both modified ITT and PP analyses. We did not find significant interaction effects for any of the outcome variables, except for fasting glucose levels. The PP analyses revealed that changes in fasting glucose levels significantly differed by the exercise mode(p = .034); significant reductions in fasting glucose levels were seen in the combination exercise group as compared with that in the aerobic group. Specifically, an additional analysis revealed that such a significant differential effect occurred at 12 months (p = .004), and the changes in fasting glucose levels in the combination exercise group showed a liner trend (p = .014) (data not shown).

**Table 2:** Differential effects of exercise mode on markers of subclinical atherosclerosis and anthropometric and cardiometabolic profiles over time

	**Modified intention-to-treat analysis (N = 92)**				**Per-protocol analysis (N = 49)**			
	**Mean (SD)**			**P**^**a**^	**Mean (SD)**			**P**^**a**^
	**Aerobic (n = 39)**	**Resistance (n = 26)**	**Combination (n = 27)**		**Aerobic (n = 20)**	**Resistance (n = 14)**	**Combination (n = 15)**	
FMD (%)				.792				.847
3 M	11.28 (3.50)	10.32 (3.80)	11.02 (3.49)		11.51 (4.02)	10.75 (3.67)	11.32 (3.37)	
6 M	11.55 (3.80)	11.22 (4.43)	11.10 (3.40)		12.38 (4.43)	11.02 (4.39)	11.46 (3.74)	
9 M	11.08 (4.05)	10.89 (4.33)	12.41 (4.27)		11.34 (4.88)	10.05 (4.11)	13.18 (4.34)	
12 M	10.70 (3.75)	11.54 (4.99)	11.30 (4.04)		10.56 (4.15)	11.26 (5.49)	11.20 (4.09)	
PWV (m/sec)				.585				.732
3 M	7.91 (1.18)	7.62 (1.16)	8.11 (1.05)		7.51 (0.77)	7.49 (0.82)	7.81 (1.04)	
6 M	7.83 (1.31)	7.52 (1.15)	7.76 (1.04)		7.36 (0.94)	7.34 (0.75)	7.37 (0.98)	
9 M	7.94 (1.32)	7.70 (1.18)	7.96 (1.01)		7.53 (1.15)	7.60 (0.83)	7.59 (1.02)	
12 M	7.88 (1.30)	7.74 (1.17)	7.96 (0.89)		7.45 (1.00)	7.67 (0.81)	7.61 (0.80)	
IMT (mm)				.574				.379
3 M	0.71 (0.19)	0.72 (0.17)	0.66 (0.10)		0.74 (0.24)	0.72 (0.19)	0.65 (0.09)	
6 M	0.68 (0.15)	0.67 (0.14)	0.64 (0.12)		0.71 (0.18)	0.65 (0.14)	0.65 (0.13)	
9 M	0.69 (0.14)	0.69 (0.14)	0.67 (0.10)		0.73 (0.16)	0.69 (0.13)	0.67 (0.10)	
12 M	0.70 (0.14)	0.70 (0.13)	0.67 (0.11)		0.73 (0.15)	0.71 (0.11)	0.68 (0.12)	
Body weight (kg)				.539				.894
3 M	71.11 (10.69)	67.20 (9.36)	72.62 (12.06)		69.48 (11.63)	64.19 (5.59)	69.06 (8.69)	
6 M	69.48 (11.70)	65.70 (9.45)	71.30 (12.25)		67.14 (12.84)	62.71 (6.27)	66.93 (7.74)	
9 M	69.31 (11.81)	65.60 (9.29)	71.69 (12.25)		65.25 (8.75)	62.64 (6.26)	67.15 (7.87)	
12 M	69.34 (11.84)	65.79 (9.34)	71.75 (12.63)		67.23 (12.70)	62.99 (6.57)	67.25 (8.98)	
Lean body mass (kg)				.717				.637
3 M	45.41 (4.96)	44.44 (5.09)	45.94 (4.95)		44.49 (5.88)	43.50 (4.92)	44.60 (3.81)	
6 M	45.06 (5.28)	44.46 (4.90)	45.90 (4.83)		44.95 (6.34)	43.62 (4.69)	44.38 (3.53)	
9 M	45.03 (5.18)	44.30 (5.02)	45.90 (4.62)		44.25 (4.62)	43.62 (5.05)	44.36 (3.24)	
12 M	44.94 (5.14)	44.29 (4.73)	45.74 (4.76)		44.91 (6.04)	43.61 (4.48)	44.07 (3.45)	
WC (cm)				.790				.825
3 M	93.44 (8.06)	90.14 (8.04)	94.42 (11.36)		91.32 (8.00)	87.26 (4.72)	90.41 (7.11)	
6 M	91.39 (9.41)	87.87 (8.56)	92.83 (12.00)		88.59 (9.67)	84.54 (5.25)	87.46 (7.38)	
9 M	90.77 (9.82)	88.12 (8.11)	93.61 (11.68)		87.61 (8.99)	85.21 (4.68)	88.39 (6.99)	
12 M	91.27 (9.33)	88.78 (7.98)	94.09 (11.58)		89.18 (9.24)	86.44 (4.97)	89.28 (7.33)	
Systolic BP (mmHg)				.935				.838
3 M	115.36 (15.84)	113.10 (11.99)	113.94 (17.98)		114.18 (12.81)	109.07 (9.46)	110.43 (13.70)	
6 M	113.06 (15.46)	112.37 (10.23)	112.74 (17.79)		111.03 (14.66)	111.29 (12.10)	107.53 (11.93)	
9 M	114.62 (14.55)	113.21 (9.53)	112.48 (17.42)		113.33 (13.19)	110.79 (8.74)	106.60 (8.01)	
12 M	113.95 (14.41)	111.94 (10.29)	112.91 (18.64)		112.03 (12.17)	108.43 (9.49)	107.37 (12.45)	
TC (mg/dL)				.517				.689
3 M	197.11 1(44.28)	192.13 (32.70)	201.30 (34.03)		205.07 (51.68)	185.94 (28.42)	191.34 (38.60)	
6 M	195.14 (36.26)	192.95 (35.23)	202.67 (38.54)		198.05 (39.55)	186.59 (28.53)	190.07 (42.89)	
9 M	201.65 (37.10)	202.18 (40.98)	207.30 (38.77)		205.51 (40.42)	203.40 (41.73)	195.81 (43.64)	
12 M	198.86 (42.22)	197.64 (41.46)	201.83 (40.73)		202.53 (50.23)	193.34 (42.04)	185.39 (43.74)	
LDL-C (mg/dL)				.458				.300
3 M	119.22 (38.47)	114.40 (29.22)	121.88 (36.97)		128.36 (47.15)	111.60 (31.37)	110.59 (40.99)	
6 M	116.65 (30.49)	115.46 (28.85)	124.18 (35.65)		121.73 (35.34)	110.64 (27.77)	112.96 (38.75)	
9 M	121.99 (32.78)	118.19 (39.83)	129.31 (39.32)		127.99 (37.16)	117.66 (46.93)	117.65 (42.97)	
12 M	116.85 (36.74)	116.70 (39.86)	123.48 (39.58)		123.66 (44.04)	113.70 (46.65)	106.80 (39.76)	
HDL-C (mg/dL)				.973				.562
3 M	52.91 (12.29)	49.25 (12.57)	53.58 (10.09)		52.27 (12.55)	46.98 (13.77)	54.99 (11.10)	
6 M	53.74 (13.04)	53.60 q(13.93)	54.79 (10.69)		53.70 (14.45)	51.99 (16.37)	56.19 (12.54)	
9 M	54.52 (12.48)	55.68 (14.20)	56.20 (10.07)		55.78 (13.15)	56.04 (16.64)	59.19 (11.69)	
12 M	57.72 (15.15)	54.69 (13.17)	56.39 (10.35)		59.09 (14.98)	53.51 (14.94)	59.39 (11.96)	
Triglycerides (mg/dL)								.784
3 M	124.90 (59.18)	142.40 (75.42)	129.23 (100.99)		122.18 (43.31)	136.77 (96.43)	128.83 (128.67)	
6 M	123.74 (71.06)	119.47 (57.73)	118.53 (56.25)		113.06 (57.94)	119.85 (69.02)	104.58 (58.74)	
9 M	125.72 (68.76)	141.51 (91.41)	108.93 (50.78)		108.70 (44.08)	148.51 (120.31)	94.87 (52.08)	
12 M	120.88 (70.84)	131.24 (82.54)	109.78 (48.56)		97.81 (38.99)	130.63 (107.88)	96.03 (48.36)	
Fasting glucose (mg/dL)				.413				.034
3 M	88.93 (9.87)	94.54 (19.83)	89.39 (7.69)		86.40 (7.51)	93.54 (12.40)	88.52 (7.78)	
6 M	85.80 (7.90)	91.68 (19.82)	85.67 (7.47)		83.85 (6.56)	89.71 1(12.61)	82.56 (7.86)	
9 M	84.98 (9.76)	93.16 (22.15)	84.97 (8.23)		82.33 (9.23)	94.61 (17.40)	81.33 (8.53)	
12 M	89.78 (8.28)	92.84 (22.41)	86.30 (7.22)		89.26 (7.07)	93.54 (18.33)	81.91 (5.95)*	
VO_2max_ (mL/kg/min)								
3 M	31.77 (5.45)	32.55 (5.04)	32.02 (5.41)	.972	32.72 (3.92)	33.75 (4.57)	33.45 (4.91)	.052
6 M	35.05 (6.72)	34.32 (5.50)	36.36 (6.90)		36.37 (4.77)	35.59 (3.42)	39.27 (4.87)	
9 M	35.62 (6.81)	35.07 (5.89)	34.49 (5.95)		38.24 (5.48)	36.25 (5.14)	36.31 (4.17)	
12 M	34.11 (6.29)	34.61 (6.05)	34.89 (6.45)		35.41 (4.84)	35.82 (5.67)	37.04 (5.28)	

^a^P-values for significance of interaction effects between exercise group and time via the generalized estimating equation.

FMD = flow mediated dilation; PWV = pulse wave velocity; HDL-C = high-density lipoprotein cholesterol; IMT = intima media thickness; LDL-C = low-density lipoprotein cholesterol; SD = standard deviation; TC = total cholesterol; VO_2max_ = maximal oxygen consumption; WC = waist circumference.

^*^P < .05 is for a significant difference in fasting glucose levels by exercise mode at 12 months.

Table 3 summarizes the time effects for all the 3 groups on the outcome variables, based on the results of modified ITT and PP analyses. We found significant time effects for PWV (p = .048) and IMT (p = .018). We also found significant time effects for body weight (p < .001), lean body mass (p = .040), waist circumference (p < .001), total cholesterol (p = .006), LDL cholesterol (p = .020), and HDL cholesterol levels (p < .001), fasting glucose levels (p < .001), and VO_2max_ (p < .001), based on the modified ITT analysis results. In other words, regardless of the exercise modes, PWV, IMT, body weight, waist circumference, and total and LDL cholesterol levels significantly decreased over time in the diet-plus-exercise intervention; HDL cholesterol level and VO_2max_ significantly increased over time in the diet-plus-exercise intervention. Specifically, changes in body weight, waist circumference, HDL cholesterol level, and VO_2max_ primarily showed linear trends. The IMT and fasting glucose levels showed quadratic curve trends, while PWV and total and LDL cholesterol levels showed cubic curve trends, based on the modified ITT analysis results. The findings from the PP analyses almost closely matched those from the modified ITT analyses.

**Table 3:** Time effects on markers of subclinical atherosclerosis and anthropometric and cardiometabolic profiles

	**Modified intention-to-teat analysis (N = 92)**		**Per-protocol analysis (N = 49)**	
	**Mean (SD)**	**P**^**a**^	**Mean (SD)**	**P**^**a**^
FMD (mm)				
3 M	10.93 (3.57)	.676	11.23 (3.67)	.628
6 M	11.33 (3.84)		1171 (4.17)	
9 M	11.41 (4.20)		11.54 (4.57)	
12 M	11.11 (4.18)		10.93 (4.47)	
PWV (m/sec)				
3 M	7.89 (1.14)	.048^‡^	7.60 (0.87)	.055
6 M	7.72 (1.19)		7.36 (0.88)	
9 M	7.88 (1.19)		7.57 (1.00)	
12 M	7.87 (1.15)		7.56 (0.88)	
IMT (mm)				
3 M	0.70 (0.16)	.018^†‡^	0.71 (0.19)	.042
6 M	0.67 (0.14)		0.67 (0.16)	
9 M	0.68 (0.13)		0.70 (0.13)	
12 M	0.69 (0.13)		0.71 (0.13)	
Body weight (kg)				
3 M	70.45 (10.86)	<.001^*†‡^	67.84 (9.46)	<.001^*†‡^
6 M	68.95 (11.37)		65.81 (9.87)	
9 M	68.96 (11.41)		65.08 (7.84)	
12 M	69.04 (11.55)		66.03 (10.14)	
Lean body mass (kg)				
3 M	45.29 (4.97)	.040^*^	44.65 (5.01)	.153
6 M	45.14 (5.02)		44.40 (5.08)	
9 M	45.08 (4.96)		44.10 (4.29)	
12 M	44.99 (4.89)		44.28 (4.86)	
WC (cm)				
3 M	92.80 ( 9.21)	<.001^*†‡^	89.88 (7.01)	<.001†
6 M	90.82 (10.10)		87.09 (7.95)	
9 M	90.85 (10.08)		87.14 (7.26)	
12 M	91.39 ( 9.81)		88.42 (7.60)	
Systolic BP(mmHg)				
3 M	114.30 (15.41)	.245	111.57 (12.21)	.253
6 M	112.77 (14.80)		110.03 (13.00)	
9 M	113.59 (14.18)		110.43 (10.65)	
12 M	113.08 (14.69)		109.57 (11.51)	
TC (mg/dL)				
3 M	196.93 (38.15)	.006^‡^	195.40 (42.20)	.013^‡^
6 M	196.73 (36.47)		192.33 (37.43)	
9 M	203.46 (38.37)		201.86 (41.14)	
12 M	199.39 (41.15)		194.66 (45.70)	
LDL-C (mg/dL)				
3 M	118.64 (35.38)	.020^‡^	118.13 (41.36)	.034^‡^
6 M	118.52 (31.52)		115.88 (34.15)	
9 M	123.07 (36.68)		121.74 (41.40)	
12 M	118.76 (38.17)		115.65 (43.25)	
HDL-C (mg/dL)				
3 M	52.07 (11.78)	<.001^*^	51.59 (12.63)	<.001^*^
6 M	54.01 (12.54)		53.97 (14.28)	
9 M	55.34 (12.25)		56.92 (13.64)	
12 M	56.47 (13.26)		57.59 (14.06)	
Triglycerides (mg/dL)				
3 M	131.12 (77.41)	.289	128.39 (90.15)	.197
6 M	121.00 (62.72)		112.40 (60.51)	
9 M	125.25 (71.84)		115.99 (77.68)	
12 M	120.55 (68.62)		106.64 (68.35)	
Fasting glucose (mg/dL)				
3 M	90.65 (13.13)	<.001^†^	89.09 (9.52)	.034^*†^
6 M	87.42 (12.54)		85.13 (9.33)	
9 M	87.29 (14.41)		85.60 (13.13)	
12 M	89.62 (13.70)		88.23 (11.93)	
VO_2max_ (mL/kg/min)				
3 M	32.06 (5.27)	<.001^*†‡^	33.23 (4.34)	<.001^*†^
6 M	35.24 (6.44)		37.04 (4.63)	
9 M	35.14 (6.26)		37.05 (4.97)	
12 M	34.48 (6.21)		36.02 (5.15)	

^a^P-values for an equality of mean values across periods; Significance of trend tests: *Linear trend, ^†^Quadratic trend, ^‡^Cubic trend.

FMD = flow mediated dilation; PWV = pulse wave velocity; IMT = intima media thickness; HDL-C = high-density lipoprotein cholesterol; LDL-C = low-density lipoprotein cholesterol; SD = standard deviation; TC = total cholesterol; VO_2max_ = maximal oxygen consumption; WC = waist circumference.

## Discussion

The exercise mode did not have significant differential effects on FMD, PWV, and IMT in the diet-plus-exercise intervention of a 12-month weight management program. However, the exercise mode had a significant differential effect on fasting glucose levels; combination exercise lowered fasting glucose levels more effectively than aerobic exercise. Meanwhile, there were significant time effects of the diet-plus-exercise intervention, i.e., reductions in PWV and IMT in cubic and quadratic trends, respectively, and improvements in body weight; waist circumference; levels of total, LDL, and HDL cholesterol; and cardiorespiratory fitness in linear, quadratic, or cubic trends.

We found neither differential effects of exercise modes nor time effects of the diet-plus-exercise intervention on FMD in women with abdominal obesity. Several studies have previously reported the beneficial effects of both aerobic and resistance exercise on FMD. Aerobic exercise training is well known to improve FMD [30-32], although only a few studies have reported the effects of aerobic exercise on FMD in obese individuals. Watts et al. [33] reported that FMD was significantly greater among trained obese children compared with untrained obese children after 8 weeks of aerobic exercise training [33]. Similarly, either resistance or combination exercise training may be effective in improving endothelial function, although this was found in small-scale studies, thereby necessitating further confirmation [13,34,35]. In a study of 30 overweight women, Olson et al. [13] reported that FMD improved significantly in a resistance exercise group compared with a control group after a 1-year resistance training intervention [13]. Maiorana et al. [35] reported in a crossover study that a combination of aerobic and resistance exercise for 8 weeks significantly increased FMD among 16 patients with type 2 diabetes mellitus [35]. Nevertheless, only a few studies have reported the differential effects of exercise mode [36]. Kwon et al. [36] found that aerobic exercise provide greater improvement in FMD than resistance exercise among 40 overweight women with type 2 diabetes mellitus in a comparison study of exercise mode [36].

In the present study, the non-significant differential effects of exercise mode on FMD for women with abdominal obesity may be explained by the complex effects of both exercise and diet in our study design. In previous studies, the effects of exercise training combined with dietary interventions have thus far yielded mixed results among obese individuals. Hamdy et al. [37] reported an improvement in the FMD in 24 obese individuals after a 6-month lifestyle modification consisting of both energy-restricted diet and aerobic exercise training [37]. In contrast, Wycherley et al. [19] did not find any significant improvement in the FMD in 29 obese patients with type II diabetes after a 12-week caloric restriction with aerobic exercise training [19]. Such conflicting findings might be attributable to distinct characteristics of FMD from other measures of subclinical atherosclerosis such as carotid IMT. The Firefighters and their Endothelium study (2005) [38] showed that the FMD had no significant correlation with carotid IMT among middle-aged men who were at low-to-moderate risk of cardiovascular diseases [38]. FMD is a well-standardized measure of endothelial function that is considered an early manifestation of atherosclerosis [39]. Furthermore, FMD is an acute indicator of vascular function, as opposed to carotid IMT, which is a chronic indicator of vascular structural abnormalities. Therefore, changes in FMD may, in part, result from acute influences such as within-individual variations in the exposure to the complex effects of either exercise or diet (i.e., changes in body weight or cardiometabolic risk factors) when they were measured. Meanwhile, non-significant changes in the FMD found in the present study may also be explained by the normal range (≥5.5%) of the FMD values at baseline (10.8%; range, 4.0–18.0% in our data). Swift et al. [31] reported a significant improvement in the FMD in obese women with impaired FMD, but not in those with normal FMD at baseline after a 6-month aerobic exercise training program [31].

We found no differential effect of exercise mode on aortic PWV, but a significant reduction in aortic PWV with the diet-plus-exercise intervention over time. Aerobic exercise training has previously been reported to decrease aortic PWV in a few studies, although the findings were limited to healthy men. Hayashi et al. [40] reported that 16 weeks of aerobic training with moderate-intensity walking and jogging significantly decreased aortic PWV in 17 sedentary middle-aged men [40]. Collier et al. [41] found that aerobic exercise led to a decrease in aortic PWV, whereas resistance exercise led to a significant increase in aortic PWV after 4-week training among 30 middle-aged individuals with prehypertension or stage1 hypertension [41]. However, such increases in aortic PWV with resistance training need to be further clarified. In fact, we did not find any increase in aortic PWV in the resistance exercise group. Similarly, a meta-analysis by Miyachi showed that resistance training may not be associated with increases in aortic PWV in middle-aged subjects [42]. Meanwhile, our data showed that the reduction in aortic PWV peaked at 6 months in the entire intervention and rebounded after the next 6 months in the diet-plus-exercise intervention in a cubic curve trend; this trend appeared to parallel with the weight loss trend in our data. Dengo et al. [43] reported that a reduction in aortic PWV correlated with reductions in total body and abdominal adiposity among overweight and obese adults [43]. In this context, the reduction of aortic PWV may be primarily related to exercise-induced energy expenditure contributing to weight loss rather than exercise mode.

We did not find any differential effect of exercise modes on carotid IMT. Instead, we found a significant regression in carotid IMT with the diet-plus-exercise intervention as a time effect. Similarly, Spence et al. [44] reported that there was no significant differential effect of aerobic versus resistance exercise training on carotid IMT, but there was a significant time effect among 23 young healthy male subjects with 24 weeks of exercise training [44]. Dutheil et al. [17] investigated the effects of combination exercise on carotid IMT according to exercise intensity, and reported that moderate-resistance high-aerobic exercise was more effective for the regression of carotid IMT than moderate-resistance moderate-aerobic exercise [17]. Thus, we speculate that the effects of exercise training on carotid IMT may be influenced by exercise intensity but not by exercise mode.

In our findings, the regression effect of exercise training on carotid IMT (i.e., a time effect) was not linear over time, with a peak at 6 months that was not maintained to 12 months, consistent with the findings of a previous study [45]. There was little evidence for beneficial effects of a lifestyle intervention (i.e., either diet-alone or diet-plus-exercise intervention) on carotid IMT among overweight and obese individuals [45-47]. Fuentes et al. [45] reported a significant regression in carotid IMT over a 24-month follow-up with a diet-induced weight-loss intervention involving 60 obese individuals [45]. Carotid IMT is a vascular measure of structural changes in the carotid arteries, so a reduction in carotid IMT may require long-duration interventions. Thus, the underlying mechanism of these short-term changes in carotid IMT with the diet-plus-exercise intervention need to be further clarified, but we could speculate that the changes are the result of various favorable changes in the cardiometabolic risk factors that occur concomitantly with weight loss induced by the diet-plus-exercise intervention.

Finally, we found a significant differential effect of exercise mode on fasting glucose levels but not on vascular measures (i.e., FMD, PWV, and IMT); specifically, combination of aerobic and resistance exercises was significantly more effective in lowering fasting glucose levels than aerobic exercise alone in the diet-plus-exercise intervention. The beneficial effect of a combination of aerobic and resistance exercises on glucose control has been previously reported only in patients with type 2 diabetes [21,48,49] but not in overweight and obese individuals. Snowling et al. [49] concluded, from their meta-analysis, that aerobic, resistance, and combination exercises had small-to-moderate beneficial effects on glucose control in patients with type 2 diabetes; particularly, compared to aerobic exercise, combination exercise was found to lower fasting glucose levels, albeit to a small extent [49]. Meanwhile, the conflicting effects of exercise mode on fasting glucose levels versus vascular measures may be explained by different exercise-induced physiological responses. The beneficial effect of combination exercise on fasting glucose levels may be attributable to an additional beneficial effect of resistance exercise to aerobic exercise. Physiologically, similar to aerobic exercise, resistance exercise can increase glucose uptake and glycogen repletion in skeletal muscle following exercise by promoting contraction-mediated GLUT4 translocation with increased activation of AMP-activated protein kinase [50]. In this context, both aerobic and resistance exercise may provide a synergistic effect on glucose metabolism. In contrast, non-significant differential effects of exercise mode on vascular measures (i.e., PWV and IMT) may indicate that changes in vascular measures influenced by vascular tone and remodeling with exercise training are not influenced by exercise mode but instead by other components such as exercise intensity or exercise expenditure [17].

This study, to the best of our knowledge, is the first to report on the effects of exercise modes in a weight management intervention on major markers of subclinical atherosclerosis such as endothelial dysfunction, arterial stiffness, and carotid IMT. Nevertheless, this study has several limitations. First, the attrition rate in the study was 55% over 12 months, and this may have led to a bias influencing the validity of results. However, the present study was initiated and implemented as a community-based program and its attrition rate was similar to that in previous studies conducted as community-based programs [51]. Second, of the participants included in the modified ITT analysis (n = 92), 68% had a menstrual cycle. FMD may modulate in response to changing hormonal patterns during the menstrual cycle [52]. Lack of consideration of the effects of the menstrual cycle in vascular measurements might have underestimated or overestimated changes in FMD. Third, since all the participants in present study were Korean women, the results cannot be generalized to men or other ethnic groups.

## Conclusions

For women with abdominal obesity, a combination of aerobic and resistance exercise may be preferable to a single mode (i.e., aerobic or resistance exercise) for effective glucose control. Regardless of exercise mode, exercise interventions combined with dietary interventions may be beneficial for reducing the risk of subclinical atherosclerosis and cardiometabolic risk in women with abdominal obesity. Such beneficial effects of exercise training may not be differentiated by exercise mode but by other exercise components such as exercise intensity or expenditure.

## Competing interests

The authors declare that they have no competing interests.

## Authors’ contributions

The authors’ contributions were as follows: JC developed the hypothesis of this study and prepared the manuscript draft; JC, JHC, and SYJ were involved in data collection; JC and JL performed the data analyses; SYJ, AS and LEB provided expert consultation on data interpretation and helped to draft the manuscript. All authors were involved in the review and revision of the manuscript and gave approval for the final version to be published.

## Pre-publication history

The pre-publication history for this paper can be accessed here:

http://www.biomedcentral.com/1471-2261/14/82/prepub
